# A study on the correlation between systemic inflammatory markers in the local tumor immune microenvironment and Porphyromonas gingivalis infection status in patients with oral squamous cell carcinoma

**DOI:** 10.3389/fonc.2026.1747849

**Published:** 2026-07-29

**Authors:** Jie Wang, Liming Xu, Xue Cao, Langqing Liu, Xiao Tian, Yulian Fu, Qiang Qiang, Bin Ling

**Affiliations:** 1 Department of Oral and Maxillofacial Oncology Surgery, The First Affiliated Hospital (Affiliated Stomatological Hospital) of Xinjiang Medical University, Urumqi, China; 2 Institute of Stomatology, Xinjiang Uygur Autonomous Region, Urumqi, China

**Keywords:** oral squamous cell carcinoma, Porphyromonas gingivalis, systemic immune inflammation index, systemic inflammatory response index, tumor microenvironment

## Abstract

**Purpose:**

This study investigates the relationship between *Porphyromonas gingivalis* (*P. gingivalis*) infection status and preoperative levels of inflammatory markers in peripheral blood as well as the tumor immune microenvironment (TIME) in patients with oral squamous cell carcinoma (OSCC).

**Methods:**

This retrospective study enrolled 201 patients with oral squamous cell carcinoma (OSCC) and collected their relevant clinical data, preoperative peripheral blood samples, and histopathological sections. Immunohistochemical analysis was performed to assess *P. gingivalis* expression levels in the patients’ tissue samples, and the patients were divided into high-expression and low-expression groups. First, we analyzed the relationships between clinical indicators—such as tumor stage and pathological grade—and *P. gingivalis* expression levels. Additionally, systemic inflammation markers, including the systemic immune–inflammation index (SII), the neutrophil–lymphocyte ratio (NLR), the platelet–lymphocyte ratio (PLR), and the systemic inflammatory response index (SIRI), were calculated on the basis of peripheral blood test results; we analyzed the correlations of these variables with clinical indicators and *P. gingivalis* expression levels. At the same time, immunohistochemical analysis was performed on OSCC tissue samples to assess the abundance of CD4+ T cells and the expression of chemokine ligand 20 (CCL20), chemokine receptor 6 (CCR6), interleukin 10 (IL-10), FOXP3, and LAG-3 in OSCC tissue samples to assess the relationship between *P. gingivalis* expression levels and systemic inflammatory markers in peripheral blood as well as the local immunosuppressive microenvironment.

**Results:**

Patients with higher *P. gingivalis* expression levels in tumor tissue had lower tumor differentiation (*P* < 0.001) and shorter survival times (*P* < 0.001). Two systemic inflammatory markers—the SII and SIRI—were associated with tumor differentiation (SII: *P* = 0.020; SIRI: *P* = 0.004), while the SII, NLR, PLR, and SIRI were associated with the T stage (SII: *P* < 0.001; NLR: *P* = 0.003; PLR: *P* = 0.017; SIRI: *P* = 0.003); the SII and PLR were associated with the N stage (SII: *P* = 0.002; PLR: *P* = 0.046). The NLR and SIRI were associated with *P. gingivalis* expression levels (NLR: *P* = 0.026; SIRI: *P* = 0.021). Multivariate logistic regression analysis revealed that pT stage is an independent risk factor for high expression of *P. gingivalis*, exhibiting a dose-response relationship (pT2: OR = 5.83, pT3: OR = 11.69, pT4: OR = 43.66; all *P* < 0.01); poorly differentiated tumors (OR = 11.02) and lip cancer (OR = 0.27) were also independent risk factors (*P* < 0.05). Immunohistochemical results revealed that OSCC patients with higher *P. gingivalis* levels had significantly higher positive expression of CD4, FOXP3, LAG3, CCR6, CCL20, and IL-10 (CD4, FOXP3, LAG3, CCR6, IL-10: *P* < 0.0001; CCL20: *P* = 0.0006). Four systemic inflammation markers—the SII, NLR, PLR, and SIRI—were associated with the expression levels of FOXP3, CCR6, CCL20, and IL-10 in OSCC tumor tissues (*P* < 0.05); among these markers, the SII and PLR were significantly associated with LAG3 expression levels (*P* < 0.05). Furthermore, only SIRI values were associated with CD4 expression levels (*P* < 0.05).

**Conclusion:**

*P. gingivalis* alters the local immune status by modulating key immune molecules—CD4, FOXP3, CCL20, CCR6, LAG-3, and IL-10—in the OSCC tumor microenvironment. These local changes are significantly associated with systemic inflammation levels, indicating a close interaction between the local immune microenvironment and systemic inflammatory responses, both of which contribute to the development and progression of OSCC.

## 1 Introduction

Oral cancer is a common malignant tumor of the head and neck. Globally, it ranks sixth among the most common types of cancer, with over 90% of cases presenting as oral squamous cell carcinoma (OSCC) (1). The International Classification of Diseases defines oral cancer as cancer of the oral cavity and pharynx, including cancers of the lips, tongue, cheeks, gums, floor of the mouth, oropharynx, nasopharynx, and hypopharynx (2). According to the latest data from the GLOBOCAN database, in 2022, lip and oral cancers combined ranked as the 16th most common malignant tumors, with approximately 389, 485 new cases reported worldwide and approximately 188, 230 deaths. Despite significant advances in early diagnosis, surgical intervention, chemotherapy, and immunotherapy, the prognosis for oral squamous cell carcinoma (OSCC) patients remains poor, with a five-year survival rate of approximately 50% (3–5). Given the complex etiology and highly aggressive nature of OSCC, the limitations of current comprehensive treatment approaches, and the significant differences in survival rates among patients at different stages, researching and systematically elucidating the pathogenic mechanisms of this disease has become a critical step in improving the effectiveness of prevention and treatment.

The human microbiome consists of a diverse array of bacteria that play crucial roles in maintaining the balance between health and disease. These microorganisms coexist with their host in a state of equilibrium, benefiting both parties. The oral microbiome may directly or indirectly contribute to the initiation and progression of OSCC by increasing inflammation and oxidative stress (6–8). *In vitro* experiments involving specific bacteria and OSCC cell lines have identified certain oral bacterial species, such as *Porphyromonas gingivalis (P. gingivalis, P.g)*, as inducing the production of inflammatory cytokines during the cellular invasion process of OSCC (9). *P. gingivalis* can induce chronic inflammation, thereby weakening the local immune response and leading to immune evasion by tumor cells (10). In particular, *P. gingivalis* enhances the invasive properties of oral squamous cell carcinoma (OSCC) by promoting the expression of chemokines (such as CCL2 and CXCL2) and cytokines (such as IL-6 and IL-8) in OSCC cells and by recruiting myeloid-derived suppressor cells (MDSCs) to suppress the function of effector T cells (11, 12). Previous research by our group has shown that *P.g* can promote the recruitment of CCR6+ regulatory T cells (Tregs) by infecting OSCC cells, thereby creating a tumor-suppressive microenvironment and promoting the malignant progression of OSCC (13).

Studies have shown that the immune system plays a role in the development and progression of OSCC (14). The tumor microenvironment (TME) can recognize tumor cells and inhibit tumor development and progression, but it can also promote immune evasion and tumor-associated inflammation (15). In the process of oral carcinogenesis, CD4+ T cells differentiate into activated Tregs with potent immunosuppressive functions, leading to the sustained conversion of CD8+ T cells into a state of exhaustion or dysfunction (16). FOXP3+ Tregs suppress the function of NK cells and dendritic cells, thereby promoting immune evasion (17). During cancer progression, the expression of inhibitory receptors such as LAG-3 on the surface of tumor-specific T cells increases, leading to the functional exhaustion of CD8+ T cells. This prevents them from proliferating in response to antigens and results in a loss of key effector functions. The resulting immune tolerance creates multiple barriers to tumor elimination, including the infiltration of regulatory T cells into tumors, coinhibitory signaling via inhibitory receptors, and the release of inhibitory cytokines such as IL-10 (18, 19). Previous research by our group has shown that *P.g* promotes the secretion of the chemokine CCL20 by infecting OSCC cells (20). The CCL20–CCR6 axis promotes Treg cell migration and reshapes the immune microenvironment, playing a crucial role in the development of oral cancer (21, 22).

Additionally, studies have hypothesized that localized inflammatory conditions may influence systemic inflammatory states, which can be measured through peripheral blood biomarkers (23–30). The extent of inflammatory responses can be thoroughly explored through malnutrition and certain indicators, among which the counts of neutrophils, lymphocytes, monocytes, and platelets in peripheral blood are readily obtainable. Additionally, systemic ratios between the aforementioned cells, such as the neutrophil–lymphocyte ratio (NLR), platelet–lymphocyte ratio (PLR), systemic immune inflammation index (SII), and systemic inflammatory response index (SIRI), have been demonstrated to be correlated with survival in cancer patients (23, 25, 31–33). Recently, the systemic immune inflammation index (SII), which is based on neutrophils, lymphocytes, and platelets, has been proposed as a comprehensive tool that provides useful prognostic information for patients with hepatocellular carcinoma, pancreatic cancer, and germ cell tumors and has been investigated in the context of oral squamous cell carcinoma (OSCC) (34–37). Inflammatory markers can play a key role in improving the diagnostic accuracy of salivary gland tumors, particularly in distinguishing between malignant and benign pathologies (38). Another significant application of these biomarkers is in the assessment of tumor prognosis. Cheng et al. found that a high NLR value before treatment is significantly associated with a worse prognosis. The authors demonstrated that the 10-year disease specific survival rate was 68% in patients with an NLR < 2.48, decreasing to 58% in patients with a higher NLR (39). However, Kawakita et al. observed that, in a group of patients with salivary ductal carcinoma, an elevated NLR (greater than 2.5) was associated with reduced overall survival, indicating an almost two-fold mortality risk (40). Also, other parameters such as SII and SIRI were found to be statistically correlated with prognosis: specifically, the sum of SII and SIRI has been shown to independently predict patient survival after surgery (41). Systemic inflammatory states can be reflected by inflammatory markers in peripheral blood; among these, readily available systemic inflammatory markers hold prognostic value for cancer, particularly in oral squamous cell carcinoma, where composite indices such as the SII demonstrate significant potential for prognostic assessment.

This study aimed to evaluate differences in the preoperative SII, NLR, PLR, and SIRI across clinical parameters such as pathological stage and differentiation grade in a cohort of 201 OSCC patients who underwent radical surgery. Furthermore, this study innovatively explored the correlation between *P. gingivalis* infection status and peripheral blood systemic inflammatory markers, as well as local tumor immune microenvironment characteristics, with the aim of providing new insights into the pathogenesis of OSCC and the assessment of its prognosis.

## 2 Materials and methods

### 2.1 Research subjects

A total of 201 patients with oral squamous cell carcinoma who underwent surgical treatment at the First Affiliated Hospital of Xinjiang Medical University between January 2007 and December 2017 were selected. The inclusion criteria for patients were as follows: (1) Patients who met clinical diagnostic criteria for oral squamous cell carcinoma (42) and were confirmed by histopathological examination. (2) Patients whose complete preoperative serological test results and clinical and laboratory data were available. (3) Patients whose complete follow-up information was obtained via telephone or outpatient visits. (4) Patients with no history of other malignant tumors. (5) Patients who did not receive radiotherapy or chemotherapy prior to surgery. The exclusion criteria were as follows: (1) Patients whose clinical or follow-up data were incomplete. (2) Patients with concomitant systemic infectious diseases, genetic metabolic disorders, endocrine diseases, or immunodeficiency disorders. (3) Patients with cognitive impairment or a history of psychiatric disorders.

### 2.2 Clinical data collection

By reviewing hospital electronic medical records, we collected the general characteristics and clinical data of patients with oral squamous cell carcinoma, including age, sex, primary tumor location, tumor stage, degree of differentiation, and lymph node metastasis status.

### 2.3 Serological marker testing

The patient provided a 5 mL venous blood sample on an empty stomach prior to surgery for testing. Automated blood analyzers were used to measure platelet, neutrophil, lymphocyte, and monocyte levels. SII = (platelet count × neutrophil count/lymphocyte count), NLR = (neutrophil count/lymphocyte count), PLR = (platelet count/lymphocyte count), and SIRI = (neutrophil count × monocyte count/lymphocyte count).

### 2.4 Hematoxylin–eosin staining and immunohistochemical staining

(1) Paraffin tumor tissue blocks from 201 patients with oral squamous cell carcinoma were retrieved from the Department of Pathology at the First Affiliated Hospital of Xinjiang Medical University for the preparation of routine histological sections. (2) Hematoxylin–eosin staining: Hematoxylin stains chromatin within the nucleus and nucleic acids in the cytoplasm purple–blue, and eosin stains cytoplasmic and extracellular matrix components red. (3) Immunohistochemical staining: Sections were dewaxed with xylene and rehydrated with a graded ethanol series. Antigen retrieval was performed by heating the sections in sodium citrate solution. The sections were blocked with peroxidase and then blocked with 5% goat serum. Afterward, the sections were incubated at 4 °C overnight with primary antibodies against *P. gingivalis* (Dia-An, Inc., South Korea), CD4 (hs-0766R, Bioss, China), CCR6 (A00957, Boster, China), CCL20 (DF2238, Affinity Biosciences, USA), FOXP3 (ab215206, Abcam, USA), LAG-3 (16616-1-ap, Proteintech, China), and IL-10 (ab189392, Abcam, USA) (all at 1:400 dilution). After the sections were incubated with the corresponding secondary antibodies and avidin–biotin peroxidase conjugates, visualization was achieved using a DAB kit (Zhongshan Jinqiao, China). The positive area was measured using ImageJ software. Each section was quantitatively analyzed under a light microscope. First, the entire section was examined under low magnification to identify and exclude the following areas: areas of tissue necrosis, areas of obvious hemorrhage, areas with section folds or knife marks, areas with severe edge effects, and areas with extensive nonspecific background staining. Using a systematic random sampling method, five representative, nonoverlapping high-magnification fields of view were randomly selected from each section. Two independent observers each selected fields of view and counted the cells, and the average was calculated.

### 2.5 Follow-up

Follow-up was conducted via telephone, through text messages, or at outpatient visits until either January 31, 2024, or patient death. Overall survival (OS) was defined as the time from the diagnosis of oral squamous cell carcinoma until patient death or the last follow-up date.

### 2.6 Statistical analysis

Data analysis was performed with the assistance of SPSS 23.0, GraphPad Prism 9, and R 3.4.3 statistical software. The Shapiro–Wilk test was used to assess the normality of all continuous variables. Continuous variables that followed a normal distribution are expressed as the mean ± standard deviation, and comparisons between groups were performed using an independent-samples t test. Continuous variables that did not follow a normal distribution are presented as medians and quartiles; comparisons between groups were performed using the Mann–Whitney U test; Categorical variables were expressed as frequencies (percentages) and analyzed using the chi-square test or Fisher’s exact test. Variables with P < 0.05 in the univariate analysis were included in the multivariate logistic regression analysis. To address the issue of abnormal odds ratios (ORs) caused by complete separation, correction was performed using the Fissell penalty in maximum likelihood estimation. A P-value of < 0.05 was considered statistically significant.

## 3 Results

### 3.1 *P. gingivalis* infection status in tissues from OSCC patients

**Figure 1:**
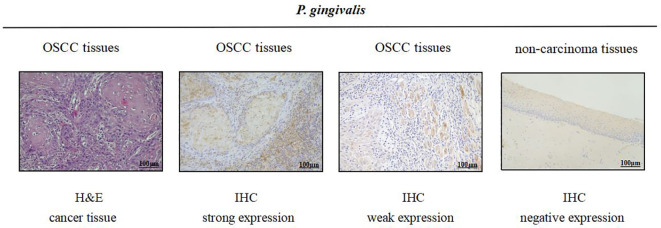
Expression of *P. gingivalis* in oral squamous cell carcinoma (OSCC) tissues.

In OSCC tissues, *P. gingivalis* immunoreactivity was strongly positive or weakly positive, as shown in **Figure 1**. After hematoxylin–eosin (H&E) staining and immunohistochemical (IHC) staining of *P. gingivalis* tissue, the IHC staining results were classified into two categories—strong expression and weak expression—based on the intensity of the immune response.

### 3.2 Correlation analysis between *P. gingivalis* infection status and clinical–pathological characteristics in OSCC patients

According to the intensity of *P. gingivalis* immunohistochemical staining in tumor tissues, members of the included cohort of 201 OSCC patients were classified into a high staining intensity group (n=118, 58.7%) and a low staining intensity group (n=83, 41.3%). To investigate the clinical significance of *P. gingivalis* infection status, we analyzed its correlations with patient baseline data and clinical pathological characteristics. Statistical analysis revealed that a high level of *P.g* was significantly associated with several key indicators (*P* < 0.05), including body weight, primary tumor site, histological grade, T stage, margin invasion, lymph node metastasis, survival status, and overall survival (see **Table 1** for details). These results indicate that a greater abundance of *P.g* in OSCC is associated with a more aggressive tumor phenotype, suggesting its potential prognostic value.

**Table 1:** Correlation between *P. gingivalis* expression levels and clinical indicators in 201 patients with oral squamous.

Variable	Low *P.g*	High *P.g*	t/Z/χ²	*p*
Total No.	83	118		
Age, mean (SD)	61.04 (12.75)	63.92 (11.54)	-1.672	0.096
Weight, mean (SD)	69.50 (17.68)	64.61 (11.67)	2.362	0.019
Gender, n (%)				
Men	38 (45.78)	63 (53.39)	0.844	0.358
Women	45 (54.22)	55 (46.61)		
Tobacco use, n (%)				
Smoker	18 (21.69)	40 (33.90)	2.970	0.085
Non-smoker	65 (78.31)	78 (66.10)		
Alcohol use, n (%)				
Drinker	12 (14.46)	24 (20.34)	0.781	0.377
Non-drinker	71 (85.54)	94 (79.66)		
Primary tumor site, n (%)				
Tongue	34 (40.96)	44 (37.29)	–	0.003
Buccal cheek mucosa	10 (12.05)	25 (21.19)		
Gingiva	10 (12.05)	28 (23.73)		
Lips	20 (24.10)	8 (6.78)		
Palate	4 (4.82)	6 (5.08)		
Floor of the mouth	3 (3.61)	4 (3.39)		
Oropharynx	0 (0.00)	3 (2.54)		
Retromolar trigone	2 (2.41)	0 (0.00)		
Histological differentiation grade, n (%)				
Well-differentiated	69 (83.13)	66 (55.93)	20.597	<0.001
Moderately differentiated	14 (16.87)	35 (29.66)		
Poorly differentiated	0 (0.00)	17 (14.41)		
AJCC pT, n (%)				
pT1	23 (27.71)	3 (2.54)	43.977	<0.001
pT2	48 (57.83)	58 (49.15)		
pT3	11 (13.25)	32 (27.12)		
pT4	1 (1.20)	25 (21.19)		
AJCC pN, n (%)				
pN0	65 (78.31)	73 (61.86)	–	0.067
pN1	6 (7.23)	18 (15.25)		
pN2	12 (14.46)	25 (21.19)		
pN3	0 (0.00)	2 (1.69)		
Local Invasion, n (%)				
Present	30 (36.14)	52 (44.07)	0.960	0.327
Absent	53 (63.86)	66 (55.93)		
Margin infiltration, n (%)				
Positive	6 (7.23)	27 (22.88)	7.596	0.006
Negative	77 (92.77)	91 (77.12)		
Lymph node metastasis, n (%)				
Present	18 (21.69)	44 (37.29)	4.853	0.028
Absent	65 (78.31)	74 (62.71)		
Clinical status at the end of the follow-up, n (%)				
Alive and without recurrence	57 (68.67)	56 (47.46)	8.071	0.004
Dead of index cancer	26 (31.33)	62 (52.54)		
Overall survival, Median [IQR]	76.00 [37.00, 112.00]	33.00 [16.00, 92.50]	3.505	<0.001

### 3.3 Determining the relationships of the SII, NLR, PLR, and SIRI with the clinical and pathological characteristics of OSCC patients

In this study, preoperative peripheral blood inflammatory markers were analyzed in 201 patients with oral squamous cell carcinoma who were undergoing radical surgery. After completing the lymphocyte, neutrophil, monocyte, and platelet counts, we calculated the systemic immune inflammation index (SII), neutrophil–lymphocyte ratio (NLR), platelet–lymphocyte ratio (PLR), and systemic inflammatory response index (SIRI). The data are summarized in **Table 2**. These findings indicate that the aforementioned inflammatory markers are significantly correlated with the clinical-pathological characteristics of multiple tumors. With respect to tumor differentiation, the SII (*P* = 0.020) and the SIRI (*P* = 0.004) were significantly associated. With respect to T stage, the SII (*P* < 0.001), NLR (*P* = 0.003), PLR (*P* = 0.017), and SIRI (*P* = 0.003) were strongly significantly associated. Notably, as T stage progressed from early (T1–T2) to advanced (T3–T4) stages, the median values of the SII, NLR, and SIRI tended to increase. With respect to lymph node metastasis (N stage), the SII (*P* = 0.002) and PLR (*P* = 0.046) were significantly associated.

**Table 2:** Association between clinicopathological variables and systemic inflammatory markers in 201 OSCC patients.

Variable	Total no.	SII	H/Z	p	NLR	H/Z	p	PLR	H/Z	p	SIRI	H/Z	p
		Median [IQR]			Median [IQR]			Median [IQR]			Median [IQR]		
Location of primary tumor													
Tongue	78	358.76 [244.95, 601.54]			2.19 [1.43, 2.76]			95.99 [76.86, 133.58]			0.96 [0.55, 1.48]		
Buccal cheek mucosa	35	384.50 [209.34, 643.08]			2.20 [1.32, 3.43]			101.10 [59.02, 135.43]			0.95 [0.54, 1.45]		
Gingiva	38	450.84 [317.33, 632.82]			2.56 [1.70, 3.53]			106.33 [85.76, 144.88]			1.09 [0.75, 1.72]		
Lips	28	368.66 [258.05, 686.45]	3.666	0.817	2.04 [1.44, 2.91]	7.620	0.367	95.00 [72.29, 134.25]	7.783	0.352	1.10 [0.55, 1.34]	8.609	0.282
Palate	10	391.82 [319.41, 497.92]			1.86 [1.34, 3.26]			96.67 [75.24, 150.60]			1.09 [0.62, 1.42]		
Floor of the mouth	7	426.82 [411.21, 755.62]			3.21 [2.36, 3.38]			155.56 [90.02, 175.82]			1.38 [1.00, 2.06]		
Oropharynx	3	448.75 [407.54, 607.15]			2.63 [2.48, 2.76]			130.83 [96.67, 177.34]			1.82 [1.49, 2.57]		
Retromolar trigone	2	444.33 [386.62, 502.04]			1.77 [1.50, 2.04]			206.73 [204.88, 208.58]			0.67 [0.54, 0.80]		
Tumor grade													
Well-differentiated	135	370.64 [243.47, 554.32]			2.19 [1.38, 2.89]			98.20 [71.32, 139.44]			0.93 [0.54, 1.35]		
Moderately differentiated	49	451.81 [295.38, 661.19]	7.858	0.020	2.45 [1.77, 3.46]	5.711	0.058	103.86 [79.49, 145.29]	3.931	0.140	1.11 [0.92, 1.86]	10.959	0.004
Poorly differentiated	17	486.82 [333.35, 1297.25]			2.37 [1.65, 4.97]			129.57 [88.58, 240.59]			1.38 [0.66, 2.78]		
AJCC pT													
pT1	26	371.12 [244.62, 457.99]			1.57 [1.27, 2.35]			99.07 [84.12, 110.52]			0.69 [0.52, 1.24]		
pT2	106	336.60 [232.24, 547.04]	19.954	<0.001	2.18 [1.46, 2.76]	13.954	0.003	91.17 [67.46, 131.86]	10.177	0.017	0.98 [0.59, 1.27]	14.118	0.003
pT3	43	535.28 [331.13, 874.29]			2.54 [1.64, 3.40]			119.87 [84.46, 189.42]			1.24 [0.74, 2.01]		
pT4	26	564.21 [369.88, 1053.39]			2.73 [2.06, 3.91]			126.78 [88.54, 171.90]			1.43 [0.95, 2.67]		
AJCC pN													
pN0	138	371.06 [247.70, 598.36]			2.20 [1.39, 3.07]			97.34 [72.00, 131.28]			0.99 [0.58, 1.41]		
pN1	24	319.70 [220.30, 505.32]	13.954	0.002	1.97 [1.58, 2.58]	7.620	0.055	101.65 [68.48, 143.80]	7.977	0.046	0.91 [0.68, 1.23]	7.043	0.071
pN2	37	572.97 [428.95, 765.55]			2.73 [1.92, 3.52]			126.42 [93.33, 175.00]			1.27 [0.95, 2.29]		
pN3	2	334.01 [279.33, 388.69]			2.01 [1.56, 2.45]			74.78 [69.94, 79.62]			1.06 [0.77, 1.34]		
Clinical status at the end of the follow-up													
Alive and without recurrence	113	425.69 [259.18, 636.70]	0.881	0.378	2.20 [1.51, 2.92]	-0.109	0.913	105.51 [79.49, 152.25]	1.419	0.156	0.97 [0.58, 1.41]	-1.126	0.261
Dead of index cancer	88	368.49 [247.83, 582.72]			2.32 [1.42, 3.22]			99.98 [69.18, 132.89]			1.11 [0.62, 1.56]		

### 3.4 Analysis of the association between *P.g* Levels and markers of systemic inflammation

Intergroup comparative analysis revealed that *P. gingivalis* levels were associated with specific systemic inflammatory markers. Patients in the high *P.g* group had significantly higher NLR (*P* = 0.026) and SIRI (*P* = 0.021) values than patients in the low *P.g* group. Numerically, the data revealed that patients in the high *P.g* group had higher SII values than patients with low *P.g*, but this result was not statistically significant (see **Table 3**).

**Table 3:** Association between *P. gingivalis* expression levels and systemic inflammatory markers in 201 OSCC patients.

Variable	Total No.	SII	H/Z	*p*	NLR	H/Z	*p*	PLR	H/Z	*p*	SIRI	H/Z	*p*
		Median [IQR]			Median [IQR]			Median [IQR]			Median [IQR]		
Low *P.g*	83	371.60 [244.98, 580.06]	-1.346	0.178	2.07 [1.38, 2.69]	-2.222	0.026	100.82 [75.59, 139.44]	-0.176	0.858	0.93 [0.58, 1.27]	-2.309	0.021
High *P.g*	118	416.34 [259.55, 643.10]			2.40 [1.57, 3.41]			101.88 [70.63, 145.82]			1.12 [0.62, 1.81]		

### 3.5 Analysis of independent risk factors for high expression of *P. gingivalis*

**Figure 2:**
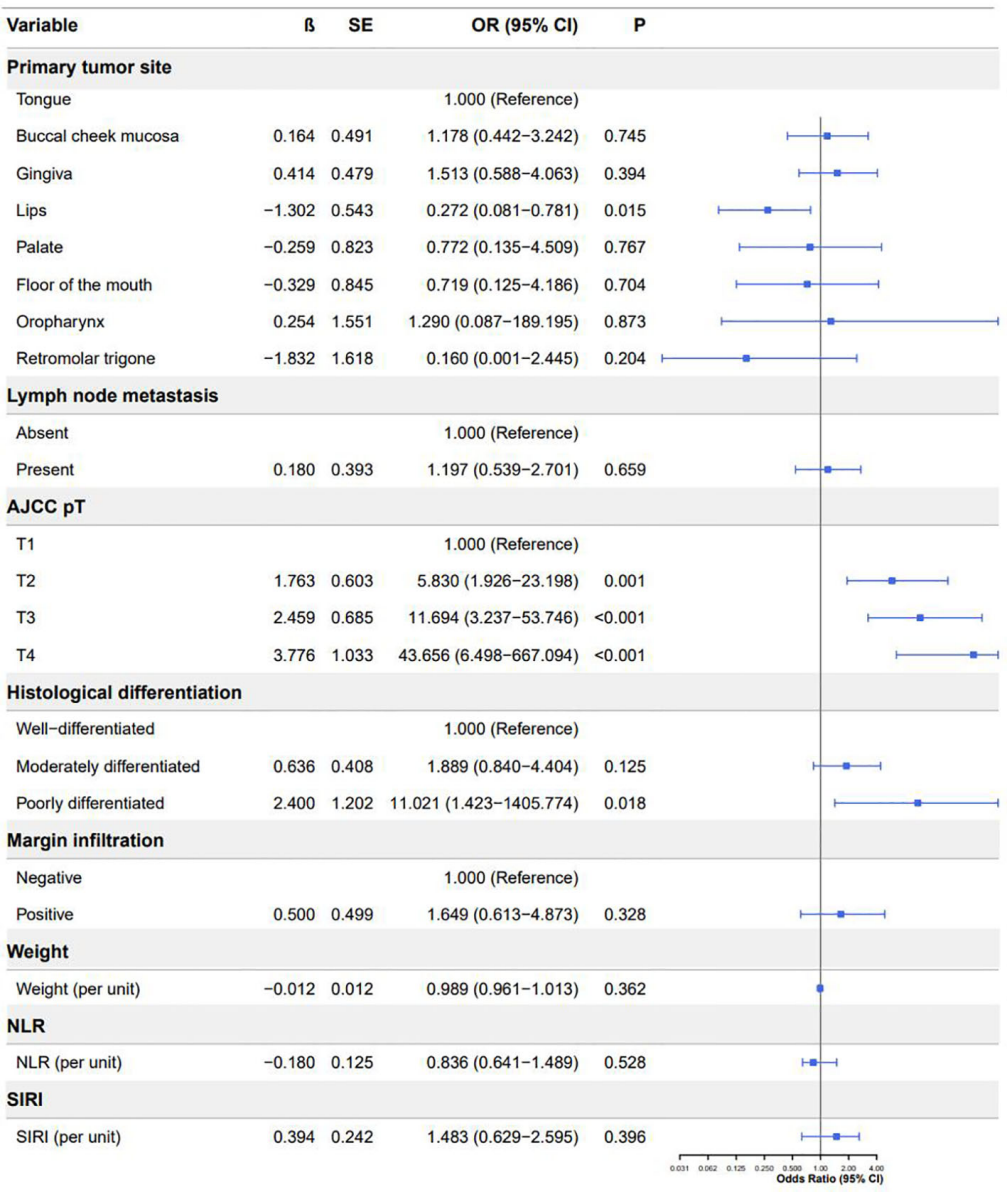
Forest plot of the multivariable logistic regression analysis. β, beta coefficient; S.E., standard error; OR, odds ratio; CI, confidence interval; Inf, infinity.

Multivariate logistic regression analysis revealed (**Figure 2**) that, after adjusting for other covariates, AJCC pT staging was found to be an independent risk factor for high expression of *P.g*. Compared with stage pT1, stage pT2 (OR = 5.830, 95% CI: 1.926–23.198, *P* = 0.001), stage pT3 (OR = 11.694, 95% CI: 3.237–53.746, *P* < 0.001), and stage pT4 (OR = 43.656, 95% CI: 6.498–667.094, *P* < 0.001) showed progressively higher odds ratios, demonstrating a clear dose-response relationship. With regard to histological differentiation, poorly differentiated tumors were significantly associated with high expression of *P.g* (OR = 11.021, 95% CI: 1.423–1405.774, *P* = 0.018). With regard to the primary site, there was a negative correlation between lip cancer and high *P.g* expression (OR = 0.272, 95% CI: 0.081–0.781, *P* = 0.015), suggesting that patients with lip cancer have lower levels of *P.g* expression. Lymph node metastasis, margin invasion, body weight, NLR, and SIRI did not reach statistical significance in the multivariate analysis (all *P* > 0.05). The confidence intervals for some variables (oropharyngeal, poorly differentiated, pT4) are relatively wide, reflecting the uncertainty resulting from the limited sample size in these subgroups.

### 3.6 Correlation analysis between *P. gingivalis* infection status and immunological characteristics of the OSCC tumor microenvironment

**Figure 3:**
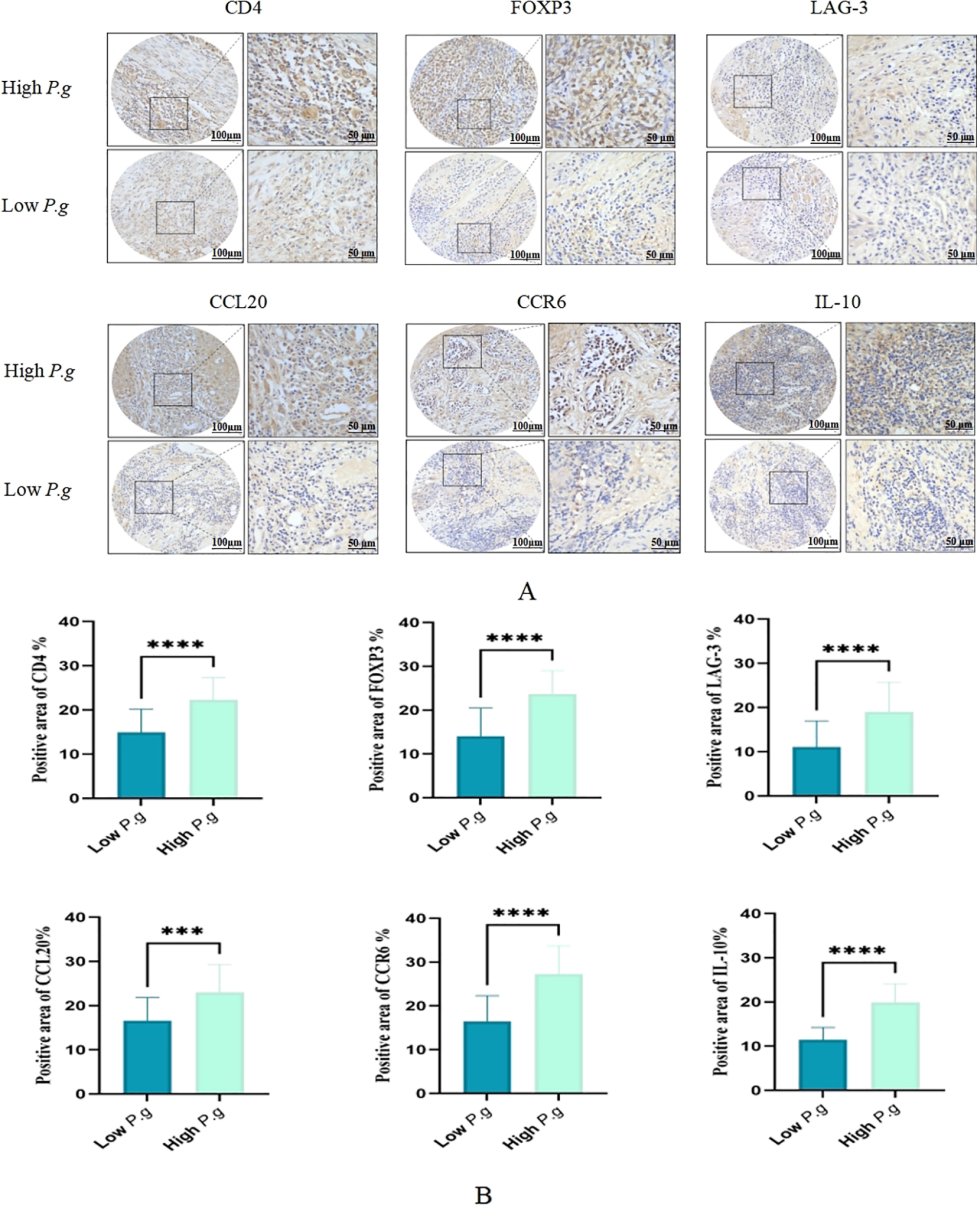
Profiles of immune cells and cytokines in the OSCC tumor microenvironment across different *P. gingivalis* levels. **(A)** Immunohistochemical analysis of CD4, FOXP3, LAG-3, CCL20, CCR6, and IL-10 expression in oral squamous cell carcinoma tissues with different levels of *Porphyromonas gingivalis*. Representative images are shown at magnifications of ×200 (scale bar = 100 μm) and ×400 (scale bar = 50 μm). **(B)** The bar graph shows the percentage of area with positive staining for different molecules in OSCC tissues with varying levels of *Porphyromonas gingivalis*. The data are presented as the mean ± SD. Statistical significance was determined by a t test: ***P < 0.001, ****P < 0.0001.

In this study, immunohistochemistry was used to quantify *P. gingivalis*, CD4, CCR6, CCL20, FOXP3, LAG-3, and IL-10 levels in 70 OSCC tissue samples (**Figure 3A**). On the basis of *P.g* levels, the sample was divided into low-level and high-level groups. Furthermore, by comparing IHC data across groups with different *P. gingivalis* levels, we evaluated the associations between the aforementioned immune markers and bacterial infection status within the tumor microenvironment. The results revealed significant differences in the expression of certain immune markers in the *P.g* high-level group. (see **Figure 3B** for details).

As shown in **Figure 3A**, among the tissue samples from 70 OSCC patients, 35 OSCC tissues exhibited weak positive staining of *P.g*, whereas 35 OSCC tissues demonstrated strong positive staining of *P.g* Compared with the *P.g* low-level group, the *P.g* high-level group showed significantly greater expression of CD4, FOXP3, LAG3, CCR6, CCL20, and IL-10. Significant differences (*P* < 0.0001) were observed in the expression of CD4, FOXP3, LAG3, CCR6, and IL-10 between the two *P.g*. groups. CCL20 expression also exhibited significant differences (*P* = 0.0006) between the *P.g* groups, as shown in **Figure 3B**.

### 3.7 Analysis of immune cells and related molecules in OSCC tissue samples in association with the SII, NLR, PLR, and SIRI

We performed a correlation analysis using the IHC data from OSCC tissue samples and systemic inflammatory marker data collected from the peripheral blood of the same OSCC patients. For this analysis, the areas positive for CD4, FOXP3, LAG3, CCR6, CCL20, and IL-10 were assessed in tumor tissues. On the basis of expression levels, the immunohistochemical markers in each group were classified into high-expression and low-expression groups and analyzed in relation to the SII, NLR, PLR, and SIRI, as shown in **Table 4**. We found that the SII and PLR had statistically significant (*P* < 0.05) associations with the expression levels of FOXP3, LAG3, CCR6, CCL20, and IL-10. Furthermore, the NLR and SIRI were significantly associated with the expression levels of FOXP3, CCR6, CCL20, and IL-10 (*P* < 0.05). Only the SIRI showed a statistically significant association with CD4 expression levels (*P* < 0.05). Thus, the data reveal several noteworthy associations between systemic inflammatory markers and the expression levels of immune cells and related molecules in the tumor microenvironment of oral squamous cell carcinoma.

**Table 4:** Association of intratumoral immune cell and molecular expression with systemic inflammatory indices in 70 OSCC patients.

Variable	SII	H/Z	*p*	NLR	H/Z	*p*	PLR	H/Z	*p*	SIRI	H/Z	*p*
	Median [IQR]			Median [IQR]			Median [IQR]			Median [IQR]		
Low CD4	335.30[251.74, 523.74]	-0.993	0.321	2.16[1.42, 2.79]	-1.473	0.140	104.25[78.07, 123.30]	-1.684	0.091	0.79[0.49, 1.17]	-2.576	0.009
High CD4	595.36[224.65, 1207.78]			2.42[1.71, 5.36]			131.86[73.06, 240.59]			1.38[0.74, 3.00]		
Low FOXP3	301.51[225.94, 523.74]	-2.559	0.010	2.01[1.50, 2.61]	-2.003	0.044	89.22[59.30, 121.22]	-3.163	0.001	0.79[0.53, 1.17]	-2.172	0.029
High FOXP3	595.36[306.31, 1207.78]			2.73[1.57, 10.93]			140.88[98.33, 266.68]			1.19[0.61, 3.20]		
Low CCR6	309.31[228.93, 464.97]	-2.332	0.021	1.92[1.20, 2.61]	-3.073	0.003	104.25[68.68, 121.22]	-2.752	0.028	0.77[0.44, 1.15]	-3.171	0.001
High CCR6	635.37[265.49, 1207.78]			2.66[1.89, 10.93]			131.86[81.03, 266.68]			1.19[0.74, 3.20]		
Low CCL20	313.90[231.82, 442.575]	-2.339	0.015	2.12[1.28, 2.63]	-2.045	0.040	94.10[66.39, 116.38]	-3.039	0.002	0.85[0.44, 1.17]	-2.517	0.011
High CCL20	635.37[260.67, 1348.86]			2.63[1.71, 10.93]			150.68[85.90, 266.67]			1.25[0.61, 3.20]		
Low LAG-3	324.42[231.82, 464.97]	-2.121	0.033	2.28[1.73, 2.65]	0.396	0.584	94.10[62.31, 123.88]	-2.480	0.015	0.96[0.57, 1.17]	-1.415	0.157
High LAG3	595.36[247.90, 1348.86]			2.63[1.43, 5.36]			129.57[88.58, 240.59]			1.25[0.58, 3.00]		
Low IL-10	313.90[241.17, 442.53]	-2.248	0.024	2.11[1.41, 2.46]	-2.635	0.008	91.60[66.39, 121.68]	-2.802	0.004	0.79[0.56, 1.16]	-2.467	0.013
High IL-10	635.37[229.44, 1348.86]			2.84[1.71, 10.93]			135.97[85.90, 266.67]			1.51[0.66, 3.20]		

## 4 Discussion

In this study, *P. gingivalis* was detected in surgical tissue samples from patients with oral squamous cell carcinoma. Further analysis of the correlation between *P. gingivalis* infection status and patients’ general characteristics and clinical indicators revealed that its levels varied across different tumor sites (such as the tongue, buccal mucosa, and gingiva). Additionally, high *P.g* levels were correlated with tumor differentiation status, with more poorly differentiated tumors exhibit significantly higher *P.g* levels in their tissue. In terms of clinical and pathological indicators, the expression levels of *P.g* are associated with clinical stage, T stage, and lymph node metastasis. Patients in the high *P.g* group were predominantly classified as T2–T3, while those in the low *P.g* group were predominantly classified as T1. The high *P.g* group had a higher proportion of patients with lymph node metastasis than the low *P.g* group. In terms of prognosis, patients in the high P.g group had shorter survival times than those in the low *P.g* group. These findings suggest that *P. gingivalis* infection may promote the progression of oral squamous cell carcinoma. Previous research has shown that high levels of *P.g* in the oral tumor microenvironment and oral tumor cells promote the epithelial–mesenchymal transition of OSCC, leading to malignant biological phenomena such as invasion and metastasis (43). The oral microbiota plays a significant role in promoting the development of OSCC by mediating the direct metabolism of chemical carcinogens and systemic inflammatory responses (10, 44). *P.g* can induce chronic inflammation, thereby weakening the local immune response and leading to immune evasion by tumor cells (45).

Porphyromonas gingivalis can also promote tumor progression by creating a carcinogenic microenvironment (46). Epidemiological studies (47) have confirmed that 16%-18% of tumors develop as a result of inflammation, indicating a relationship between inflammation and tumorigenesis. In this study, we investigated the clinical relevance of four preoperative systemic inflammatory markers and their significance in the local immune microenvironment of OSCC. According to our findings, the SII and SIRI are correlated with tumor differentiation status. Oral squamous cell carcinoma varies in its degree of differentiation—low, moderate, and high—and the corresponding SII and SIRI values also differ accordingly. An analysis of 201 OSCC patients in this study revealed significant differences in the SII, NLR, PLR, and SIRI across primary tumor size, regional lymph node involvement, and distant metastasis. Regarding tumor differentiation, the SII and SIRI were significantly associated with tumors of varying differentiation levels; overall, the levels of inflammatory markers tended to be higher in the poorly differentiated tumor group, suggesting that poorer tumor cell differentiation may be accompanied by a more pronounced systemic inflammatory state. In terms of T stage, SII, NLR, PLR, and SIRI significantly strongly differ across the T stages. Furthermore, as T stages progressed from early to advanced stages, the median values of the SII, NLR, and SIRI tended to increase together. These findings indicate that the greater the degree of local tumor invasion is, the higher the overall level of inflammation in the patient’s body. Regarding lymph node metastasis, the SII and PLR emerged as significantly correlated. Specifically, the PLR typically indicates elevated platelet counts (thrombocytosis) and reduced lymphocyte counts in blood samples (lymphopenia), potentially reflecting an imbalance in immune system responses that impairs adequate control over tumor cells (48). The data revealed that patients with regional lymph node metastasis had significantly higher levels of inflammatory markers than those without lymph node metastasis did, suggesting that systemic inflammatory responses may influence a tumor’s ability to metastasize to lymph nodes.

These findings suggest that the SII, NLR, PLR, and SIRI may serve as potential biomarkers for assessing OSCC disease progression. Mechanistically, inflammatory responses participate in tumorigenesis and progression by modulating relevant blood components. Therefore, monitoring the dynamic changes in the SII, NLR, PLR, and SIRI may provide valuable information to facilitate the early diagnosis of OSCC. Recent studies have indicated that the NLR comprehensively reflects the inflammatory and immune status of tumor patients (49), thereby reflecting the balance between protumor inflammation and antitumor immune responses (50). In this study, by evaluating the NLR and SIRI among OSCC patients at different stages, we observed that both measures increased significantly with advancing T stages in OSCC patients. Recent studies have revealed that both the NLR and the PLR are significantly elevated in patients with underlying malignant diseases and oral cancer (51). Findings over the past decade have linked elevated NLR and PLR with poor prognosis across various types of malignant tumors (52). The SII and SIRI are newly proposed prognostic biomarkers. In lung cancer patients, a high SII is correlated with a low survival rate (53). A high SII and SIRI are correlated with poor disease-specific survival (DSS) in oral squamous cell carcinoma (OSCC) patients, making them promising prognostic biomarkers for OSCC (54). In this study, our data revealed associations between the NLR and the SII and between tumor size and clinical stage, which is consistent with the previously noted promotion of tumor growth and spread. An elevated NLR and SII may result from neutrophilia, thrombocytosis, and lymphopenia, thus reflecting the combined effects of inflammation and immune system dysfunction.

In this study, by analyzing the relationships between *P.g* levels and systemic inflammatory markers in OSCC patients, we revealed that *P.g* levels were strongly correlated with the NLR and the SIRI. NLR and SIRI values increased as *P.g* levels increased in the tumor tissues of OSCC patients, suggesting that the local immune response triggered by *P.g* infection may further influence systemic immune status. Furthermore, immunohistochemical analysis of OSCC tumor tissues revealed increased expression of CD4, CCR6, and its ligand CCL20 in the P.g high-level group. Concurrently, the expression of the immunoregulatory molecules FOXP3, LAG-3, and IL-10 was significantly more pronounced. These findings suggest that *P. gingivalis* infection plays a crucial role in local immune regulation in OSCC. CD4+ T cells are central to adaptive immunity, and the significant activation of the CCR6–CCL20 axis suggests that *P. gingivalis* infection may result in the recruitment of many CCR6+ immune cells (such as Th17 cells and Tregs) to the tumor site (55, 56). Notably, we observed increased expression of FOXP3—a key Treg transcription factor—alongside the immune checkpoint molecule LAG-3 and the immunosuppressive cytokine IL-10, collectively establishing a highly immunosuppressive microenvironment: FOXP3+ Tregs and IL-10 directly suppress the antitumor function of effector T cells, while LAG-3 further depletes T-cell activity (57). Increased FOXP3+ expression indicates more extensive T-cell infiltration; in this context, the cytotoxic function of CD8+ T cells outweighs the inhibitory effect of Tregs, and there is an association between a higher CD8+/FOXP3+ ratio and a more favorable prognosis (58). This multimolecular, multipathway immunosuppressive state likely represents a key mechanism by which *P. gingivalis* promotes tumor immune evasion. *P. gingivalis* can directly induce Treg differentiation and IL-10 production through its virulence factors (such as gingipain) (59).

Our data show that the SIRI is associated with the level of CD4+ expression in tumors. In addition, the SII and PLR are significantly associated with the expression of FOXP3, LAG-3, and CCR6, and the NLR and SIRI are also associated with the expression of FOXP3 and CCR6. High expression of FOXP3 and IL-10 is significantly associated with all four inflammatory markers, suggesting that a state of local immune suppression coexists with a systemic proinflammatory state. In other words, chronic systemic inflammation may drive simultaneous local immune suppression, and the two may jointly promote tumor progression. High expression of CCR6 and CCL20 was also significantly associated with all inflammatory markers. The CCL20/CCR6 axis is a key chemotactic pathway for the recruitment of Tregs and Th17 cells to the tumor site. The activation of this axis may simultaneously promote the formation of a locally immunosuppressive microenvironment and amplify the systemic inflammatory response, thereby serving as a key link between local and systemic immune–inflammatory states. High expression of LAG-3 is associated only with the SII and PLR, which may suggest that T-cell exhaustion is more closely linked to platelet-associated inflammatory pathways and less closely linked to granulocyte and monocyte pathways. High CD4 expression was significantly associated only with the SIRI, which integrates data on neutrophils, monocytes, and lymphocytes. This finding suggests that an increase in total CD4+ cells may be associated with more complex dysregulation of myeloid immune cells rather than a generalized inflammatory response.

In addition to the CCL20–CCR6 axis, *P.g* may also reshape the tumor immune microenvironment of OSCC through other mechanisms. First, regarding the TLR signaling pathway, *P.g* can activate Toll-like receptor 4 (TLR4) through lipopolysaccharide (LPS) components in outer membrane vesicles (OMVs), selectively inducing monocytes to develop tolerance to tumor necrosis factor (TNF). Moreover, this effect is partially mediated by the synergistic interaction between mTOR signaling and IL-10, thus impairing the host’s ability to effectively clear *P.g* (60). In addition, *P.g* interferes with the recognition of the TLR4 signaling pathway by modifying the structure of lipopolysaccharide A, thereby disrupting the balance between innate and adaptive immune responses and promoting systemic inflammation (61). Regarding metabolic reprogramming, *P.g* may interfere with chemotherapy-induced cell death by inhibiting ceramide-dependent mitochondrial autophagy, thereby not only making OSCC cells resistant to treatment but also potentially influencing the infiltration and function of immune cells indirectly through the regulation of the metabolic state of tumor cells (62). Therefore, the TLR signaling pathway and metabolic reprogramming may act in concert with the CCL20–CCR6 axis to promote the malignant progression of OSCC.

These experimental results indicate that systemic inflammatory states are closely associated with local immune suppression in OSCC. This may be due to the systemic inflammatory response leading to increased circulating neutrophils and platelets, thereby reducing the number of lymphocytes available to combat tumor cells (63). The significant association between the SIRI and CD4 expression may be the result of systemic inflammatory states affecting T-cell recruitment or their fate within the local tumor microenvironment (64). FOXP3+ Tregs are known to be key immunosuppressive cells, whereas CCR6 serves as a crucial receptor that mediates the migration of immune cells to tumor sites. Its ligand, CCL20, is frequently produced by tumor cells or myeloid cells under inflammatory stimulation (55). CCL20 levels continue to increase in OSCC tissue and saliva, with a particularly marked increase once the disease progresses to the advanced stage, and it exhibits functional characteristics that promote tumor progression (65). Systemic inflammatory markers are significantly correlated with the proportion of FOXP3+ Tregs and CCR6 expression, suggesting that systemic inflammatory states may actively recruit or induce immunosuppressive Tregs to accumulate at tumor sites via the CCL20–CCR6 axis, thereby shaping a microenvironment conducive to immune evasion. The SII as well as PLR and LAG-3 expression levels in tumor tissues are significantly correlated, suggesting that systemic inflammation may be associated with the establishment of T-cell exhaustion states (57). Cancer progression is well known to require interactions between tumor cells and their microenvironment, including inflammatory, immune, and metabolic responses. Consequently, immune cells perform distinct functions locally and systemically, promoting cancer initiation and progression (37).

This study is a retrospective study and may be subject to information bias and selection bias. Furthermore, regional, ethnic, and clinical practice-specific factors may be at play, so caution is warranted when generalizing these findings. Future validation will require large-scale, multicenter, prospective cohort studies. This study covered a relatively long-time span. Although the diagnostic criteria and principles of curative surgery remained consistent throughout this period and we excluded patients who received adjuvant therapy prior to surgery, we cannot completely rule out potential confounding biases associated with the passage of time. Owing to limitations in sample size, the current study was unable to fully establish a causal relationship. These findings require validation using larger-scale clinical data, and mechanistic investigations are needed to clarify the role of specific biological pathways. This study focused solely on preoperative static inflammatory conditions; data regarding dynamic changes during surgery and related diagnostic efficacy require further in-depth analysis and systematic organization. Future research should integrate comprehensive, real-time monitoring data to fully assess the potential value of such data for supporting treatment decisions and monitoring disease progression.

## 5 Conclusion

In summary, our findings suggest that high expression of Porphyromonas gingivalis in oral squamous cell carcinoma tissues is one of the key factors influencing its malignant progression. *P.g*. infection is associated with elevated levels of systemic inflammation, suggesting that *P.g*. may indirectly promote the progression of OSCC by exacerbating the body’s systemic inflammatory response. At the tumor site, the infection status of *P.g* reshapes the immune microenvironment of OSCC. The level of infection is closely associated with changes in the expression of several key immune markers in the tumor microenvironment, suggesting that this bacterium may influence tumor progression by modulating the local immune response. In the progression of OSCC, there is a close and complex interaction between systemic inflammatory responses and the local tumor immune microenvironment, which may collectively constitute the pathological basis for *P.g*.’s promotion of tumor progression.

## Data Availability

The original contributions presented in the study are included in the article/supplementary material. Further inquiries can be directed to the corresponding author.
